# Use of Platelet-Rich Fibrin and Platelet-Rich Plasma as Delivery Systems for Natural Compounds: A Systematic Review

**DOI:** 10.3390/ma19142970

**Published:** 2026-07-10

**Authors:** Bartosz Chwaliszewski, Wojciech Niemczyk, Małgorzata Kępa, Izabela Nawrot-Hadzik, Rafał Wiench, Robert Wojtyczka, Michał Ciszyński, Amelie Lupp, Marzena Dominiak, Jakub Hadzik

**Affiliations:** 1 Medical Center of Innovation, Faculty of Dentistry, Wroclaw Medical University, Krakowska 26, 50-425 Wroclaw, Poland; 2 Department of Dental Surgery, Faculty of Dentistry, Wroclaw Medical University, Krakowska 26, 50-425 Wroclaw, Poland; 3 Department of Periodontal Diseases and Oral Mucosa Diseases, Faculty of Medical Sciences in Zabrze, Medical University of Silesia, pl. Traugutta 2, 41-800 Zabrze, Poland; 4 Department of Microbiology, Faculty of Pharmaceutical Sciences in Sosnowiec, Medical University of Silesia in Katowice, Jagiellońska 4, 41-200 Sosnowiec, Poland; 5 Department of Pharmaceutical Biology and Biotechnology, Faculty of Pharmacy, Wroclaw Medical University, ul. Borowska 211, 50-556 Wroclaw, Poland; 6 Institute of Pharmacology and Toxicology, Jena University Hospital, Friedrich Schiller University Jena, Drackendorfer Straße 1, D-07747 Jena, Germany

**Keywords:** platelet-rich fibrin, platelet-rich plasma, natural compounds, drug delivery, regenerative dentistry, tissue regeneration, biomaterials

## Abstract

Background/Objectives: Natural compounds have gained increasing attention in regenerative dentistry and medicine because of their antimicrobial, anti-inflammatory, antioxidant, and pro-healing properties. However, their clinical translation is often limited by poor stability, low bioavailability, and insufficient local retention. Platelet-rich plasma (PRP) and platelet-rich fibrin (PRF) may help overcome these limitations by functioning as autologous scaffolds or local delivery systems. This systematic review evaluated the available evidence on the use of PRP and PRF as carriers, scaffolds, or delivery matrices for natural compounds in regenerative medicine and tissue healing. Methods: A systematic search was performed in PubMed, Scopus, and Embase from database inception to 1 April 2026, in accordance with PRISMA guidelines. Results: The search identified 113 records, of which 11 studies met the inclusion criteria. Six studies investigated PRP-based systems and five investigated PRF-based systems. Eleven studies were included: three in vitro or ex vivo biomaterial studies, three studies combining in vitro and animal experiments, three animal studies, and two clinical studies. The natural compounds evaluated included icariin, curcumin, tannic acid, propolis, tea polyphenols, gallic acid, oligomeric procyanidins, and Moringa oleifera. The strongest direct carrier evidence was observed for freeze-dried PRP loaded with icariin, which demonstrated sustained release and improved regenerative outcomes. Most PRF-based studies focused on scaffold modification or adjunctive use rather than strict carrier function. Overall, the included studies suggested favorable effects on scaffold properties, local delivery behavior, wound healing, and tissue regeneration, but the evidence was dominated by preclinical studies and showed a moderate-to-high risk of bias. Conclusions: PRP and PRF appear to be promising autologous platforms for the local delivery of natural compounds. However, the current evidence remains limited, heterogeneous, and largely preclinical, and well-designed clinical studies are needed before routine clinical application can be recommended.

## 1. Introduction

Dentistry has increasingly moved toward biologically driven and minimally invasive therapeutic strategies that can improve healing while reducing treatment-related morbidity. In this context, natural compounds have attracted substantial interest because they exhibit broad antimicrobial, anti-inflammatory, antioxidant, analgesic, and antimutagenic activities, and may offer fewer and milder adverse effects than many synthetic agents. Recent dental research has therefore focused on plant-derived bioactives such as curcumin, quercetin, allicin, rosmarinic acid, and eugenol for applications ranging from plaque control and periodontal therapy to oral mucosal disease and supportive cancer care [1,2,3,4].

At the same time, autologous platelet concentrates have become important biomaterials in regenerative dentistry. Platelet-rich plasma (PRP) and platelet-rich fibrin (PRF) are recognized as autogenous sources of growth factors involved in tissue healing and regeneration, and their use has expanded across oral surgery, periodontology, implantology, and regenerative endodontics [5,6]. Their biological rationale is based on the ability to concentrate and deliver platelets, leukocytes, cytokines, and growth factors into the wound environment, thereby supporting angiogenesis, soft-tissue repair, and hard-tissue regeneration. Platelet-based delivery systems can be prepared in different technological forms, including injectable liquids, fibrin gels, solid membranes, freeze-dried matrices, sponges, and composite hydrogels. The selected form determines how the natural compound is incorporated, retained, protected, and released at the application site. Injectable formulations may be delivered directly into soft tissues, whereas membranes and sponges are generally placed within wounds or surgical defects. Topical application to oral mucosa may also be possible using mucoadhesive gels, films, or pastes; however, such systems must resist salivary dilution, mechanical displacement, and rapid clearance from the gingival surface [7,8,9,10].

PRP is considered a first-generation platelet concentrate, whereas PRF is a second-generation product characterized by the formation of a fibrin matrix without the need for anticoagulants [11,12,13]. Compared with PRP, PRF may provide a more sustained release of growth factors because these mediators are retained within a fibrin network and released gradually over time [14,15,16].

Despite the therapeutic promise of natural compounds, their clinical translation is often constrained by physicochemical and pharmacokinetic limitations [17]. Many natural molecules show encouraging in vitro and preclinical effects [18,19], but these findings are not always reproduced in clinical settings because of inadequate stability, poor solubility, limited bioavailability, and insufficient local retention at the target site [20]. Curcumin is a representative example, as its hydrophobic nature limits its bioavailability and has led to the development of multiple delivery systems such as nanoemulsions, nanomicelles, liposomal formulations, and other encapsulation approaches [21]. More broadly, recent reviews in dentistry have emphasized that low solubility, instability, and bioavailability remain major obstacles for the clinical use of natural biomaterials and that improved delivery strategies are needed to overcome these disadvantages [22].

These considerations make platelet concentrates particularly attractive as potential carrier systems for natural compounds [23]. Beyond their intrinsic regenerative potential, PRP and PRF can function as scaffolds or biologically active matrices, offering local retention, clot stability, and a microenvironment rich in signaling molecules relevant to tissue repair [24]. In dentistry, PRP has already been described as a scaffold for regeneration, and PRF membranes have been highlighted for their gradual release of growth factors, angiogenic support, antibacterial potential, and ability to stabilize graft materials [25,26]. Taken together, these properties suggest that PRP and PRF may serve not only as regenerative adjuncts but also as delivery platforms capable of enhancing the local therapeutic performance of natural bioactive compounds.

Although the literature separately supports the use of natural compounds in dentistry and the use of PRP/PRF in regenerative procedures, the specific role of platelet concentrates as carriers or delivery matrices for natural compounds has not been clearly synthesized. This is an important question because it sits at the intersection of two rapidly developing areas: natural bioactive therapeutics and autologous regenerative biomaterials. A focused synthesis is therefore needed to determine how PRP and PRF have been used in association with natural compounds, whether they have functioned as true carriers or merely as adjunctive matrices, and what regenerative outcomes have been reported. Accordingly, the aim of the present systematic review was to evaluate the available evidence on the use of PRF and PRP as carriers, scaffolds, or local delivery systems for natural compounds in dentistry and regenerative medicine.

## 2. Materials and Methods

### 2.1. Review Design and Reporting Standard

This systematic review was conducted to evaluate the available evidence on the use of platelet-rich fibrin (PRF) and platelet-rich plasma (PRP) as carriers, scaffolds, or local delivery systems for natural bioactive compounds in regenerative medicine and tissue healing. The review was designed and reported in accordance with the Preferred Reporting Items for Systematic Reviews and Meta-Analyses (PRISMA) guidelines (The PRISMA Checklist can be found in the Supplementary Materials).

The review protocol was registered in the International Prospective Register of Systematic Reviews (PROSPERO; registration number CRD420261370384).

### 2.2. Review Question

The review addressed the following questions: what evidence exists regarding the use of PRF or PRP as carriers or delivery matrices for natural bioactive compounds, and whether these combinations improve regenerative or healing outcomes when compared with PRF/PRP alone, the natural compound alone, or other control interventions.

For the purposes of this review, a delivery-platform study was defined as an investigation in which PRF or PRP directly contributed to the loading, incorporation, immobilization, retention, permeation, or controlled release of a natural compound and in which at least one delivery-related property was evaluated. Studies examining changes in the physical or biological properties of PRF/PRP after the addition of a natural compound were classified as scaffold-modification studies. Studies in which PRF/PRP and a natural compound were administered together but only regenerative or clinical outcomes were assessed were classified as combination-therapy studies rather than true carrier studies.

### 2.3. PICOS Framework

The eligibility criteria were structured according to the PICOS framework.

(P)opulation: The population included in vitro studies, animal models, and human subjects involved in tissue repair, wound healing, bone regeneration, periodontal regeneration, oral and maxillofacial regeneration, tendon–bone healing, nerve regeneration, and other regenerative medicine applications.

(I)ntervention: The intervention consisted of PRF or PRP used in combination with a natural bioactive compound, where PRF/PRP served as a carrier, scaffold, membrane, injectable matrix, reservoir, or local delivery system. Natural compounds included plant-derived compounds, phytochemicals, plant extracts, bee-derived products, and other naturally derived bioactive substances.

(C)omparator: The comparator groups included PRF or PRP alone, the natural bioactive compound alone, placebo or no treatment, conventional regenerative treatment, or alternative biomaterial or drug-delivery systems.

(O)utcomes: The primary outcomes included evidence of carrier or delivery function, such as loading capacity, release kinetics, retention, permeation, and sustained-release behavior, as well as regenerative efficacy. Secondary outcomes included histologic, radiographic, biomechanical, cellular, molecular, and clinical healing outcomes, in addition to biocompatibility, cytotoxicity, and adverse events.

(S)tudy design: Original research studies, including in vitro studies, animal studies, randomized clinical trials, and non-randomized clinical studies, were considered eligible.

### 2.4. Eligibility Criteria

Studies were included if they evaluated PRF, including its variants such as L-PRF, A-PRF, c-PRF and i-PRF, or PRP in combination with at least one natural bioactive compound. Studies were also required to assess regenerative, healing, biological, or release-related outcomes. Only original research articles were included.

Studies were excluded if they investigated PRF or PRP without any natural compound, focused exclusively on synthetic drugs or non-natural additives, or were review articles, editorials, letters to the editor, conference abstracts without sufficient data, expert opinions, or case reports. Studies with inadequate methodological detail or insufficient outcome data were also excluded.

### 2.5. Information Sources and Search Strategy

A comprehensive electronic search was performed in PubMed, Scopus and Embase from database inception until 1 April 26. The search strategy was developed around three core concepts: platelet concentrates, carrier or delivery function, and natural bioactive compounds. In PubMed, controlled vocabulary and free-text terms were combined using MeSH and Title/Abstract field tags. In Scopus, advanced field codes were used to search the title, abstract, and keyword fields. In Embase, advanced search syntax was applied using title, abstract, and keyword fields, and controlled vocabulary was incorporated when appropriate. The reference lists of all included studies and relevant review articles were also screened manually to identify additional eligible records.

PubMed, Embase, and Scopus were selected to provide complementary biomedical, pharmacological, and multidisciplinary coverage of both clinical and preclinical research. Web of Science was not included because of its substantial overlap with Scopus, whereas the Cochrane Library primarily indexes controlled clinical evidence and was considered less likely to identify the predominantly preclinical and biomaterial studies relevant to the review question. Nevertheless, the omission of these databases may have resulted in missed records and is acknowledged as a limitation.

The search terms included synonyms and related expressions for platelet-rich fibrin and platelet-rich plasma, together with terms describing carrier function, delivery systems, scaffolds, matrices, and natural bioactive compounds such as phytochemicals, plant extracts, flavonoids, polyphenols, curcumin, icariin and propolis. In PubMed, MeSH terms were used where available, including Platelet-Rich Plasma, Phytochemicals, Plant Extracts, Curcumin, and Propolis, in combination with free-text keywords to improve sensitivity. The search terms for the relevant databases, along with the number of search results, are presented in Table 1.

### 2.6. Study Selection

All retrieved records were exported into reference management software, and duplicate entries were removed. Two reviewers independently screened the titles and abstracts of all identified studies. Full texts of potentially relevant articles were then assessed independently according to the predefined inclusion and exclusion criteria. Any disagreements were resolved through discussion, and when necessary, consultation with a third reviewer. The study selection process was documented using a PRISMA flow diagram.

### 2.7. Data Extraction

A standardized data extraction form was developed and applied to all included studies. Two reviewers independently extracted the relevant data. The extracted information included author name, year of publication, country, study design, experimental model or patient population, type of platelet concentrate, type and source of natural compound, method of incorporation into PRF/PRP, comparator groups, target tissue or application, follow-up period, outcome measures, principal findings, and reported adverse effects or complications.

### 2.8. Risk of Bias Assessment

The methodological quality and risk of bias of the included studies were assessed independently by two reviewers using tools appropriate for each study design. Randomized clinical trials were assessed using the RoB 2 tool, non-randomized studies were assessed using ROBINS-I, and animal studies were evaluated using the SYRCLE risk-of-bias tool. Risk-of-bias judgments were based on methodological domains specified by the respective assessment tools and were not determined by the magnitude, direction, or statistical significance of the reported treatment effects. Disagreements between reviewers were resolved by discussion and consensus.

### 2.9. Data Synthesis

A qualitative synthesis of the included studies was performed. The findings were summarized and compared descriptively according to the type of platelet concentrate used, the type of natural bioactive compound, the mode of incorporation or delivery, and the regenerative application investigated. The results were organized narratively because of the expected heterogeneity among studies in terms of study design, experimental model, intervention characteristics, outcome measures, and follow-up periods. No meta-analysis was performed.

## 3. Results

### 3.1. Results of Study Selection

**Figure 1:**
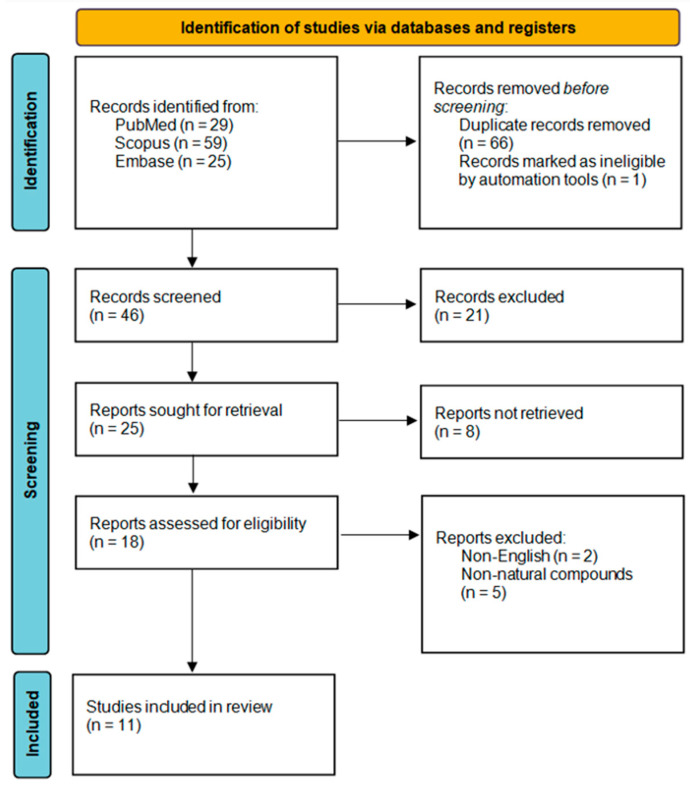
PRISMA 2020 flow diagram [27].

The database search retrieved 113 records before deduplication, including 29 from PubMed, 59 from Scopus, and 25 from Embase. After screening and eligibility assessment, 11 studies were included in the qualitative synthesis. The process for including a publication is illustrated in Figure 1 below.

### 3.2. Study Characteristics

A total of 11 studies were included. Three studies were in vitro or ex vivo biomaterial investigations, three combined in vitro and animal experiments, three were animal studies, and two were clinical human studies. Six studies investigated PRP-based systems [28,29,30,31,32,33] and five investigated PRF-based systems [34,35,36,37,38]. The natural compounds examined across the included studies were icariin, curcumin, tannic acid, propolis, tea polyphenols, gallic acid, oligomeric procyanidins, and Moringa oleifera. The studies were highly heterogeneous with respect to platelet concentrate preparation, formulation strategy, target tissue, comparator groups, and outcome measures.

Most studies were preclinical and focused on scaffold modification, controlled release, wound repair, or local regenerative enhancement. Only two studies were conducted in human participants, both in dental applications. One of these assessed 20% propolis irrigation as an adjunct to PRF in gingival recession surgery, whereas the other compared PRF alone with PRF combined with Moringa oleifera or simvastatin around dental implants. The main characteristics of the included studies, including study design, platelet concentrate type, natural compound, application model, and comparator groups, are summarized in Table 2.

The included studies used several distinct technological approaches. These comprised icariin incorporated into freeze-dried PRP, natural-polyphenol-containing injectable PRP hydrogels, curcumin-loaded nanostructured carriers combined with an L-PRF-wrapped sponge, tannic-acid-modified PRF membranes or scaffolds, and adjunctive formulations such as nano-propolis ointment or propolis irrigation. Accordingly, the routes of application ranged from implantation and injection to placement within wounds or surgical sites. None of the included studies directly demonstrated sustained topical delivery of a natural compound across intact gingiva.

### 3.3. Synthesis of the Included Evidence

The included studies clustered into two broad study categories. The first category consisted of direct carrier or scaffold-engineering studies, in which PRF or PRP was incorporated into a modified biomaterial system to improve loading, retention, membrane stability, or controlled release. This study category was represented by the icariin-loaded FD-PRP study by Zheng et al. [28], the L-PRF-associated curcumin/metronidazole nanocomposite by Murgia et al. [34], the PRP hydrogels containing tea polyphenols and gallic acid by Zhao and Yuan [29], the OPC-containing PRP hydrogel by He et al. [31], and the two tannic-acid-modified PRF studies by Haghparast-Kenarsari et al. [38] and Wang et al. [35].

The second category consisted of adjunctive therapeutic studies, in which a natural compound was used together with PRF or PRP to enhance regenerative outcomes rather than to demonstrate a formal delivery platform. This study category was seen in the studies combining PRP with curcumin-preconditioned stem cells, nano-propolis, curcumin in nerve repair, propolis irrigation with PRF in recession surgery, and PRF with Moringa oleifera around implants. Table 3 shows the main findings of the included studies.

### 3.4. Results of PRP-Based Studies

Among the PRP-based studies, the clearest evidence for PRP functioning as a true carrier was provided by Zheng et al. In that rabbit tendon–bone healing model, FD-PRP was used as a sustained-release carrier for icariin, and the ICA/FD-PRP group showed superior radiologic, histologic, and mechanical healing compared with FD-PRP alone and saline control [28].

Two recent PRP-hydrogel studies also emphasized controlled or prolonged release. Zhao and Yuan developed an injectable self-healing EOPM hydrogel that combined PRP with tea polyphenols, gallic acid, and metal–phenol nanoparticles for infected diabetic wounds; the system reduced inflammation, enhanced angiogenesis, and accelerated wound repair in a diabetic infected wound model [29]. He et al. developed a ROS/pH dual-responsive PRP-loaded hydrogel containing oligomeric procyanidins and reported sustained release of total growth factors, PDGF, TGF-β1, and FGF together with faster wound closure, reduced inflammation, and increased angiogenesis in a full-thickness mouse wound model [31].

The remaining PRP studies primarily showed adjunctive benefit. Ghufran et al. found that curcumin-preconditioned hASCs co-transplanted with PRP improved diabetic wound closure, neovascularization, fibroblast proliferation, and expression of healing markers in rats [30]. Wafy et al. reported that PRP combined with nano-propolis produced superior modulation of oxidative stress and improved tissue repair in a canine wound model compared with controls [32]. Zavala et al. showed that PRP plus curcumin produced better sciatic functional index, electrophysiological recovery, axonal counts, and myelin thickness than PRP alone, curcumin alone, or repair alone in rats with acute sciatic nerve injury [33].

### 3.5. Results of PRF-Based Studies

The PRF-based literature was more heterogeneous. Murgia et al. described a multifunctional nanocomposite sponge containing curcumin nanostructured lipid carriers and metronidazole that was intended to be used together with L-PRF in post-extraction sockets. The study demonstrated curcumin accumulation, metronidazole permeation through L-PRF and porcine buccal tissue, and acceptable cytocompatibility, supporting the feasibility of an L-PRF-associated delivery platform rather than a standalone PRF-only carrier [34].

Two PRF studies focused on tannic acid as a natural scaffold modifier. Haghparast-Kenarsari et al. reported that tannic acid crosslinking improved PRF scaffold structure, reduced swelling and degradation, increased mechanical strength, and preserved cell proliferation and antibacterial activity [38]. Wang et al. similarly found that tannic-acid-modified H-PRF membranes exhibited improved mechanical performance, prolonged degradation resistance, enhanced antibacterial activity, and maintained cytocompatibility [35].

The two clinical PRF studies suggested potential adjunctive benefit in dentistry. Bora et al. reported that 20% propolis irrigation combined with PRF in microsurgical pouch-and-tunnel treatment of Miller class I and II recession defects produced significantly greater reduction in recession depth and greater gain in keratinized tissue width than PRF alone at 3 months [36]. Balani et al. found that PRF combined with Moringa oleifera was associated with less crestal bone loss than PRF alone around dental implants, although the study also included a non-natural simvastatin arm and reported comparable improvement in the PRF + SIM and PRF + MO groups [37]. Importantly, the study did not adequately characterize the Moringa oleifera intervention, as the type of extract, plant part used, method of preparation, source of the material, and mode of incorporation into PRF were not reported. This limits reproducibility and weakens interpretation of the observed effect.

### 3.6. Overall Pattern of Findings

Taken together, the included studies suggested that the evidence base was dominated by preclinical investigations and that direct carrier evidence was limited to a small number of studies. The most convincing direct carrier study was the FD-PRP–icariin model. Several additional biomaterial studies supported the broader concept that PRF or PRP could be integrated into natural-compound-containing scaffolds or modified by natural polyphenols to improve stability, release behavior, antibacterial performance, or local regenerative effects. The clinical literature remained sparse and was restricted to small dental studies with short follow-up. No meta-analysis was performed because of marked heterogeneity in study design, target tissue, formulation strategy, comparator groups, and reported outcomes.

### 3.7. Risk of Bias Assessment Results

Risk of bias was assessed according to the methodology prespecified in the review. The randomized clinical trial was appraised using RoB 2, the non-randomized comparative human study was appraised using ROBINS-I principles, and the animal studies were appraised using a SYRCLE-based judgment framework. The three in vitro or ex vivo biomaterial studies were not formally scored because these instruments are not intended for bench-only studies; instead, they were narratively appraised for methodological limitations such as lack of randomization, absence of blinding, exploratory design, and limited translational generalizability.

Overall, the included evidence showed a moderate-to-high risk of bias profile. No study was judged to be unequivocally low risk across all domains. The randomized clinical trial by Bora et al. was judged as having some concerns rather than low risk, because although randomization, allocation concealment, blinding, sample-size calculation, and complete follow-up were reported, the article contained internal inconsistencies in group labeling and intervention description. The non-randomized study by Balani et al. was judged to have serious risk of bias because randomization, concealment, and blinding were not reported and the methodological description of group assignment was limited.

The overall judgments reflected limitations in study design and reporting rather than the statistical significance of individual outcomes. A statistically significant result was therefore not considered evidence of low risk of bias, and a non-significant result was not considered evidence of high risk of bias.

Among the animal studies, the risk of bias was usually driven by incomplete reporting of allocation concealment, housing procedures, and blinded outcome assessment. Zheng et al. reported random allocation, random specimen selection, and semiquantitative histology by two independent investigators, which supported a judgment of some concerns rather than high risk. Zavala et al. also reported random allocation and complete follow-up, but did not clearly report assessor blinding, so the study was likewise judged as having some concerns. Studies such as the PRP/nano-propolis canine wound model were considered at higher risk because multiple wounds were created within the same animals, creating potential unit-of-analysis bias and possible pseudoreplication, in addition to limited reporting of masking procedures. Risk of bias assessment is summarized in Table 4 below.

## 4. Discussion

### 4.1. Summary of Main Findings

This systematic review found that the evidence for using platelet-rich fibrin and platelet-rich plasma as carriers for natural compounds was promising but still preliminary. The strongest direct carrier evidence in the included literature came from the freeze-dried PRP–icariin study, in which PRP clearly functioned as a sustained-release matrix and was associated with improved regenerative outcomes. By contrast, most PRF-based studies did not test “carrier function” in a strict pharmacotechnical sense; instead, they evaluated PRF as a scaffold, membrane, or adjunctive biologic combined with a natural compound. Overall, the evidence suggested a consistent signal toward enhanced healing, improved scaffold properties, or improved local biologic performance, but the certainty of evidence remained low because the field was dominated by preclinical studies, small samples, and heterogeneous methods [28,34,37].

A second key finding is that the current literature appears to include two related but distinct concepts: true carrier/delivery studies and adjunctive combination studies. This distinction was important. In true carrier studies, the platelet concentrate itself contributed to local retention, release control, or scaffold-based delivery. In adjunctive studies, PRF or PRP and the natural compound were simply used together, often with positive regenerative effects, but without direct evidence that the platelet concentrate controlled release or drug availability [28,29,31,34,35,38]. The existing body of evidence therefore supported biological plausibility more strongly than it supported firm clinical efficacy [12].

### 4.2. Interpretation of Results

The principal finding of this review is not that PRF and PRP have already been established as delivery systems for natural compounds, but that the literature comprises two methodologically distinct groups. A limited number of studies directly evaluated delivery-related properties, whereas most studies investigated combined or adjunctive biological effects without demonstrating loading, retention, or controlled release. This distinction is essential because improved healing after co-administration does not itself confirm that PRF or PRP functioned as a carrier.

These findings suggested that platelet concentrates may be valuable not only because they contain endogenous growth factors, leukocytes, and fibrin, but also because their structure may be inherently suitable for local drug delivery. PRF forms a dense three-dimensional fibrin network and releases growth factors over time, while liquid forms such as i-PRF provide a short working window before clot formation, allowing bioactive substances to be mixed into the matrix before gelation [23]. Mechanistically, this creates a rational basis for local delivery: the fibrin mesh can entrap a compound, slow diffusion, and combine the effect of the loaded agent with the regenerative signaling already present in the platelet concentrate [9].

Within the natural-compound literature included in this review, this mechanistic rationale was most convincingly demonstrated for FD-PRP carrying icariin [28]. In that model, improved tendon–bone healing likely resulted from two overlapping mechanisms: first, prolonged local exposure to a plant-derived osteogenic compound; and second, the intrinsic reparative environment generated by platelet-derived growth factors [28]. In the PRF literature, a similar principle was suggested, but often indirectly. Tannic acid enhanced membrane strength and degradation resistance, curcumin-containing systems supported local permeation and tissue regeneration, and propolis- or Moringa-containing combinations suggested beneficial adjunctive effects in soft- and hard-tissue healing [34,35,36,37,38]. Thus, the review does not simply indicate that “natural compounds work” or that “PRF/PRP works”; rather, it suggests that combining both may create a multifunctional microenvironment that supports release, scaffold stability, antimicrobial control, and tissue repair simultaneously [28,34,36,37,38].

An important contextual point was that the broader PRF literature outside the natural-compound field already supported PRF as a drug-carrier platform. A 2025 systematic review of antibiotic-loaded PRF identified 13 in vitro studies, found mostly moderate risk of bias, and concluded that antimicrobial efficacy depended on the antibiotic, concentration, and loading method; it also noted that PRF often outperformed collagen sponges as a local antimicrobial carrier. Recent in vitro and clinical studies further suggested that PRF or i-PRF can carry clindamycin, triple-antibiotic formulations, ampicillin/sulbactam, or ciprofloxacin, with evidence of sustained release or improved local antimicrobial performance [23].

The same broader trend was now extending beyond antibiotics. In the attached literature, tranexamic acid incorporated into PRF increased clot and membrane weight, improved tear resistance, and was measurably entrapped within PRF, suggesting that PRF can also function as a carrier for hemostatic agents [39]. In addition, a 2025 in vitro study showed that c-PRF and liquid-phase concentrated growth factor could carry voriconazole and fluconazole without impairing clot formation; although the antifungal results were selective and less robust than the antibiotic literature, the study still supported the concept that APCs can serve as vehicles for localized antifungal delivery [40]. Taken together, these data strengthen the interpretation that PRF is best understood as a platform technology rather than as a single-purpose regenerative membrane [39,40].

### 4.3. Limitations

This review has several important limitations. First, the included studies were highly heterogeneous with respect to platelet-concentrate subtype, centrifugation protocol, loading method, natural compound, tissue target, comparator, and outcome reporting. This heterogeneity prevented quantitative synthesis and limited direct comparison across studies. Second, the evidence base was dominated by preclinical and bench studies. Only two human studies were identified, both in dentistry, and both were small. As a result, the clinical applicability of the findings remains uncertain. Third, the methodological quality of the included evidence was limited. The randomized clinical trial was judged to have some concerns, the non-randomized human study had serious risk of bias, and most animal studies lacked full detail on allocation concealment, assessor blinding, or other design safeguards.

The marked heterogeneity of the included studies substantially limits the interpretation and generalizability of the findings. The studies differed in experimental level, target tissue, PRF/PRP preparation, natural compound, incorporation method, comparator, outcome measure, and follow-up period. Consequently, results from in vitro biomaterial studies, animal models, and clinical investigations cannot be interpreted as estimates of a common treatment effect. Bench studies mainly demonstrate physicochemical feasibility, whereas animal experiments provide model-specific biological proof of concept and may not predict clinical performance in humans. Moreover, findings obtained in cutaneous wounds, tendon–bone regeneration, or peripheral nerve repair cannot be directly extrapolated to periodontal or implant-related applications because these tissues differ in vascularity, mechanical environment, healing dynamics, and therapeutic objectives. Therefore, the present review maps the range of potential applications but does not establish general effectiveness across regenerative indications.

An additional limitation concerns the absence of a formal methodological-quality assessment for the in vitro and biomaterial components of the evidence. The risk-of-bias tools applied to clinical and animal studies do not adequately assess laboratory-specific issues such as biological and technical replication, blinding of outcome assessment, standardization of platelet-concentrate preparation, validation of loading and release methods, or selective reporting of experimental conditions. Consequently, the reported risk-of-bias findings should not be interpreted as an appraisal of the entire evidence base. Positive bench-study findings should instead be regarded as preliminary demonstrations of technical feasibility and biological plausibility, particularly where independent replication and comprehensive physicochemical characterization were lacking.

The included in vitro and ex vivo studies also had limitations that were especially relevant for a carrier-focused question. Many studies showed that a compound could be mixed with PRF/PRP and that healing or antimicrobial outcomes improved, but relatively few rigorously characterized loading efficiency, release kinetics, stability of the active molecule within the fibrin matrix, or the effect of loading on endogenous PRF/PRP growth-factor release. In other words, a number of studies supported “combination efficacy,” but fewer truly established “carrier pharmacology.” This distinction was one of the major methodological weaknesses of the current field.

Another important limitation is the incomplete phytochemical and pharmaceutical characterization of some natural-compound interventions. In particular, in the study using Moringa oleifera with PRF, the authors did not report the type of extract, plant part, preparation method, source of the material, or method of incorporation into the platelet concentrate. Such omissions limit reproducibility, prevent meaningful comparison with other studies, and reduce confidence in the biological interpretation of the findings.

There are also limitations in the review process itself. The search was restricted to selected databases and to English-language articles, which may have excluded relevant reports from other sources. No meta-analysis was performed because of the marked heterogeneity noted above. In addition, the review question sat across dentistry, wound healing, tissue engineering, and regenerative medicine, so some degree of conceptual overlap was unavoidable during screening, especially when deciding whether a study represented a true carrier model or only a combined regenerative approach. These factors should be taken into account when interpreting the conclusions.

### 4.4. Comparison with Existing Literature

The present findings are consistent with broader review-level evidence indicating that PRF and PRP have wide regenerative applications in dentistry, but that the strength of evidence varies substantially by indication and many conclusions remain limited by study quality [7,12]. A recent umbrella review on PRF and PRP in dentistry concluded that the field showed considerable clinical potential, but also emphasized the need for more well-designed randomized trials before clearer clinical guidance can be issued [12]. Our review aligned with that conclusion, but narrowed the focus to one specific translational question: whether platelet concentrates can act as carriers for natural compounds. On that narrower question, the evidence was much thinner than the general regenerative literature [41].

At the same time, our findings are strongly aligned with the emerging drug-delivery literature on PRF. A recent systematic review of antibiotic-loaded PRF suggested that PRF is a plausible localized carrier for conventional antimicrobials, although protocol variability and moderate in vitro bias were major concerns [40]. Recent studies also suggested that PRF-based carriers can be extended to antifungals and to hemostatic agents such as tranexamic acid [39,40]. Therefore, when our natural-compound findings are viewed alongside the broader carrier literature, a coherent pattern emerges: the biologic and structural characteristics of PRF appear suitable for use as a local reservoir across multiple drug classes, but robust clinical translation is still lagging behind proof-of-concept experimentation [42,43].

This broader literature also helped explain why the present review found stronger support for PRF/PRP as a platform than for any single natural compound. Conventional drug-carrier studies in PRF tend to use explicit release assays [44,45,46,47,48], inhibition-zone testing, and carrier comparisons, whereas many natural-compound studies still prioritize regenerative endpoints alone [28,31,34]. As a result, the natural-compound field remains one step behind the antibiotic-carrier field methodologically. That difference should be viewed not as a contradiction, but as an indicator of where future research needs to mature.

### 4.5. Implications for Practice and Policy

From a clinical standpoint, the current evidence did not justify routine adoption of PRF/PRP-natural compound combinations as standardized care pathways. The most defensible interpretation was that these strategies should still be regarded as promising adjunctive or investigational approaches rather than replacements for established surgical, periodontal, endodontic, or wound-healing protocols. In particular, clinicians should be cautious about extrapolating from preclinical carrier studies directly to chairside practice, because dose, release kinetics, scaffold behavior, and regulatory classification remain insufficiently standardized.

However, the review does support a practical implication for clinicians already using platelet concentrates: PRF and PRP may be more than passive biologic adjuncts. They may serve as customizable local delivery matrices when a clinical scenario calls for site-specific therapy and when reducing systemic exposure is desirable. This concept may be especially relevant in oral surgery, regenerative periodontics, infected wound sites, and potentially lesions with mixed inflammatory-microbial biology. Even so, any such use should currently be framed within experimental protocols, institutional oversight, or carefully documented off-label practice rather than broad routine use.

At a policy level, the evidence points toward the need for clearer translational pathways for autologous drug-loaded biomaterials. Standardization of PRF/PRP preparation, reporting of rotor type and relative centrifugal force, loading technique, and compound concentration should be encouraged in both journals and trial protocols. Without these details, reproducibility will remain poor and clinical uptake will remain fragmented.

### 4.6. Implications for Future Research

This review was undertaken because natural compounds show considerable regenerative potential but are frequently limited by poor stability, solubility, bioavailability, and local retention, whereas PRF and PRP may provide autologous fibrin-based matrices capable of localized delivery. The gap analysis demonstrated that direct evidence of carrier performance remains scarce, as most studies assessed biological outcomes without quantifying compound loading or release. Future research should therefore prioritize loading efficiency, release kinetics, local retention, preservation of compound bioactivity, and formulation reproducibility, with loading and release characteristics representing the most fundamental properties required to confirm a true delivery function.

Future studies should move beyond simple “combination” designs and adopt genuine drug-delivery frameworks. At minimum, this should include standardized reporting of PRF/PRP subtype, tube material, centrifugation parameters, timing of compound addition, loading efficiency, release kinetics, and effects on fibrin architecture and endogenous growth-factor release. Comparative studies should directly test whether PRF/PRP offers advantages over other local carriers, rather than assuming superiority. In vivo work should be followed by randomized human trials with clinically meaningful endpoints, adequate sample-size justification, and blinded outcome assessment.

A particularly worthwhile future direction is the investigation of natural bioactive compounds with already emerging evidence in oral wound healing, periodontal regeneration, or oral inflammatory modulation as PRF/PRP-delivered agents. Current literature already points to several promising classes of phytochemicals for PRF/PRP-based delivery strategies, including curcumin, epigallocatechin gallate (EGCG), berberine, flavan-3-ols/proanthocyanidins, and stilbene derivatives such as resveratrol or piceid (polydatin). These compounds have demonstrated varying combinations of antioxidant, anti-inflammatory, antimicrobial, osteogenic, and fibroblast-supporting effects relevant to oral wound healing and periodontal regeneration [49,50,51,52]. Candidate compounds should preferably be selected based on prior evidence of compatibility with oral cell types, effects on periodontal inflammation or biofilm modulation, and relevance to oral wound-healing biology rather than solely on general systemic pharmacologic activity.

For natural compounds in general, a rational research program would begin with formulation studies in i-PRF, c-PRF, and solid PRF membranes, comparing free compounds, PRF-loaded formulations, and appropriate reference controls. The first endpoints should be loading capacity, chemical stability in the fibrin matrix, release kinetics, and any effect on clot formation or membrane mechanics. The second tier should assess biologic performance in gingival fibroblasts, keratinocytes, periodontal ligament cells, osteoblast-lineage cells, and macrophage polarization models. Because many natural compounds exhibit dose-dependent or context-dependent effects, future studies should also include careful concentration optimization, cytotoxicity testing, and comparisons between free and carrier-bound formulations.

Subsequent preclinical studies should then test natural-compound-loaded PRF/PRP in biofilm-challenged and inflammatory models rather than in sterile defects alone. Because future carrier studies should reflect real oral disease settings, they should include multispecies biofilms, patient-derived microbial isolates where feasible, and mechanically relevant outcome measures. This recommendation is reinforced by the recent antifungal-carrier literature, which explicitly noted that reference strains and laboratory conditions may not capture clinical behavior and called for work on wild-type patient-derived isolates and more realistic release models.

Natural compounds may also have potential in nonsurgical gingival augmentation. Clinical studies suggest that injectable platelet-rich fibrin and hyaluronic acid can increase gingival thickness, while i-PRF may provide outcomes comparable to free gingival grafting with less postoperative discomfort [24]. Given their pro-proliferative and regenerative properties, natural compounds may enhance the effects of nonsurgical gingival augmentation by stimulating fibroblast activity, collagen synthesis, and tissue remodeling. Their incorporation into i-PRF could therefore potentiate gingival thickening, although this hypothesis requires confirmation in clinical studies.

The safety and quality control of PRF/PRP as a carrier also require specific consideration. Although autologous preparation reduces the risks of immune incompatibility and disease transmission, it does not eliminate risks associated with venipuncture, microbial contamination during chairside handling, premature platelet activation, or substantial variability in the final product. Product characterization should therefore include the initial blood count, final platelet, leukocyte and erythrocyte concentrations, total volume, activation method, anticoagulant where applicable, and the interval between blood collection and centrifugation [53,54,55]. Centrifuge model, rotor geometry, relative centrifugal force, processing time, and collection-tube material should also be reported because these variables may influence cellular distribution, fibrin architecture, mechanical properties, and growth-factor release. Only sterile, single-use, medical-grade systems suitable for therapeutic preparation should be used, as silica-coated diagnostic tubes may introduce silica microparticles into PRF matrices and raise cytotoxicity concerns [56,57]. For compound-loaded preparations, quality control should evaluate the final combined product, including clotting time, fibrin structure, mechanical integrity, loading reproducibility, release kinetics, preservation of endogenous growth-factor release, compound stability and bioactivity, and process-related sterility or bioburden. Additional donor eligibility, infectious-disease screening, traceability, and batch-release requirements would be necessary for allogeneic or cord-blood-derived platelet products. The absence of these standardized carrier-specific assessments in most included studies represents an additional barrier to clinical translation [58].

Several translational barriers must be addressed before PRF- or PRP-based delivery of natural compounds can be considered for routine clinical use. First, natural origin does not guarantee safety, and potential cytotoxicity, allergenicity, contamination, dose-dependent effects, and interactions between the compound and platelet-derived mediators require systematic evaluation. Second, botanical products may vary according to species, plant part, cultivation conditions, extraction method, purity, and active-compound content, making source characterization and batch-to-batch standardization essential. Quality-control requirements should therefore include standardized PRF/PRP preparation and loading procedures, sterility and contaminant testing, compound identity and concentration, stability, loading reproducibility, and validated release profiles. Regulatory classification may also be complex because these preparations combine an autologous biologic material with a bioactive compound. Finally, donor-dependent variability, limited processing and handling times, chairside preparation, training requirements, cost, storage limitations, and difficulty in scaling a personalized autologous product may restrict practical implementation. These challenges reinforce that the available evidence represents an early proof of concept rather than readiness for routine clinical application.

Taken together, the available findings support the biological plausibility and technical feasibility of combining PRF or PRP with natural compounds. However, they do not establish clinical effectiveness, as most evidence was derived from in vitro or animal studies and only two small clinical studies were identified. Moreover, improvements in regenerative outcomes may reflect synergistic biological effects rather than a specific delivery function; therefore, the translational relevance of these approaches remains uncertain and requires confirmation in well-designed clinical trials.

## 5. Conclusions

The available evidence indicates that PRF and PRP can be combined with natural compounds and may contribute to favorable scaffold characteristics or regenerative responses in preclinical models. However, direct evidence of loading, retention, and controlled delivery is limited, and most included studies evaluated adjunctive biological effects rather than a true carrier function. Given the heterogeneity of the studies, the lack of formal quality assessment for bench investigations, and the presence of only two clinical studies, the findings primarily support biological plausibility and technical feasibility rather than clinical effectiveness. PRF- and PRP-based delivery of natural compounds should therefore remain investigational until standardized formulation studies and well-designed clinical trials are available.

## Figures and Tables

**Table 1:** Search terms for the relevant databases, along with the number of search results.

Database	Search Terms	Number of Records
PubMed	((“Platelet-Rich Plasma”[Mesh] OR “platelet-rich plasma”[tiab] OR PRP[tiab] OR “platelet-rich fibrin”[tiab] OR PRF[tiab] OR “leukocyte platelet-rich fibrin”[tiab] OR “advanced platelet-rich fibrin”[tiab] OR A-PRF[tiab] OR “injectable platelet-rich fibrin”[tiab] OR i-PRF[tiab]) AND (“Drug Delivery Systems”[Mesh] OR carrier*[tiab] OR “delivery system*”[tiab] OR scaffold*[tiab] OR matrix[tiab] OR matrices[tiab] OR “sustained release”[tiab] OR “controlled release”[tiab] OR loading[tiab] OR permeation[tiab] OR reservoir*[tiab]) AND (“Phytochemicals”[Mesh] OR “Plant Extracts”[Mesh] OR “Propolis”[Mesh] OR “Curcumin”[Mesh] OR phytochemical*[tiab] OR “plant extract*”[tiab] OR flavonoid*[tiab] OR polyphenol*[tiab] OR “natural compound*”[tiab] OR “natural bioactive*”[tiab] OR curcumin[tiab] OR icariin[tiab] OR propolis[tiab]))	29
Scopus	TITLE-ABS-KEY ( (“platelet-rich fibrin” OR “platelet rich fibrin” OR PRF OR “platelet-rich plasma” OR “platelet rich plasma” OR PRP OR “leukocyte platelet-rich fibrin” OR “advanced platelet-rich fibrin” OR “injectable platelet-rich fibrin”) AND (carrier* OR “delivery system*” OR scaffold* OR matrix OR matrices OR “drug delivery” OR “sustained release” OR “controlled release” OR loading OR permeation OR reservoir*) AND (phytochemical* OR “plant extract*” OR flavonoid* OR polyphenol* OR “natural compound*” OR “natural bioactive*” OR curcumin OR icariin OR propolis)	59
Embase	( (‘platelet rich fibrin’:ti,ab,kw OR ‘platelet-rich fibrin’:ti,ab,kw OR prf:ti,ab,kw OR ‘platelet rich plasma’:ti,ab,kw OR ‘platelet-rich plasma’:ti,ab,kw OR prp:ti,ab,kw OR ‘leukocyte platelet rich fibrin’:ti,ab,kw OR ‘advanced platelet rich fibrin’:ti,ab,kw OR ‘injectable platelet rich fibrin’:ti,ab,kw) AND (carrier*:ti,ab,kw OR ‘delivery system*’:ti,ab,kw OR scaffold*:ti,ab,kw OR matrix:ti,ab,kw OR matrices:ti,ab,kw OR ‘drug delivery’:ti,ab,kw OR ‘sustained release’:ti,ab,kw OR ‘controlled release’:ti,ab,kw OR loading:ti,ab,kw OR permeation:ti,ab,kw OR reservoir*:ti,ab,kw) AND (phytochemical*:ti,ab,kw OR ‘plant extract*’:ti,ab,kw OR flavonoid*:ti,ab,kw OR polyphenol*:ti,ab,kw OR ‘natural compound*’:ti,ab,kw OR ‘natural bioactive*’:ti,ab,kw OR curcumin:ti,ab,kw OR icariin:ti,ab,kw OR propolis:ti,ab,kw) )	25

**Table 2:** Characteristics of the included studies.

Author and Year	Design/Model	Platelet Concentrate	Natural Compound(s)	Application/ Formulation	Main Comparator(s)
Zheng et al., 2019 [28]	Controlled laboratory animal study; rabbit partial patellectomy model	Freeze-dried PRP	Icariin	Icariin incorporated into FD-PRP as a sustained-release carrier for tendon–bone healing	FD-PRP alone; saline control
Zhao and Yuan, 2024 [29]	In vitro + animal biomaterial study; S. aureus-infected diabetic mouse wound model	PRP	Tea polyphenols, gallic acid	EOPM hydrogel composed of pullulan derivatives, PRP, and metal–phenol nanoparticles	Hydrogel composition groups and control conditions
Ghufran et al., 2020 [30]	In vitro + animal study; STZ-induced diabetic rat excisional wound model	PRP	Curcumin	Curcumin-preconditioned hASCs co-transplanted with PRP	Saline; PRP alone; hASCs + PRP
Murgia et al., 2020 [34]	In vitro/ex vivo formulation study	L-PRF	Curcumin (with metronidazole co-loaded in the nanocomposite)	Hyaluronate-based sponge with curcumin NLCs and metronidazole designed to be wrapped in L-PRF for post-extraction sockets	Empty sponge/formulation comparisons; permeation through L-PRF and porcine buccal tissue
Wafy et al., 2026 [32]	Prospective animal study; experimental canine cutaneous wounds	PRP	Nano-propolis	Single peri-lesional PRP with or without nano-propolis ointment in lanolin carrier	Control; lanolin; nano-propolis; PRP; PRP + lanolin; PRP + nano-propolis
Wang et al., 2025 [35]	In vitro biomaterial study using donor-derived H-PRF membranes	Horizontal PRF	Tannic acid	H-PRF membrane modified with tannic acid to improve membrane performance	Unmodified H-PRF and different TA conditions
Haghparast-Kenarsari et al., 2024 [38]	In vitro scaffold study	PRF	Tannic acid	Tannic-acid crosslinked PRF scaffold for wound-healing applications	Non-crosslinked PRF and different TA concentrations
He et al., 2024 [31]	In vitro + animal biomaterial study; full-thickness mouse wound model	PRP derived from human cord blood	Oligomeric procyanidins	ROS/pH-responsive CMCS/Odex/OPC/PRP hydrogel with sustained GF release	Control and non-PRP hydrogel groups
Bora et al., 2026 [36]	Randomized controlled clinical trial; 18 patients, 29 recession sites	PRF (L-PRF)	Propolis	20% propolis irrigation as adjunct to pouch-and-tunnel surgery with PRF	PRF-based surgery without propolis
Balani et al., 2024 [37]	Human comparative clinical study; 36 implants	PRF	Moringa oleifera extract (type of extract, plant part, and preparation method not reported)	PRF scaffold combined with Moringa oleifera extract; the method of extract preparation, source, and incorporation into PRF was not specified by the authors	PRF alone; PRF + simvastatin
Zavala et al., 2023 [33]	Controlled animal study; rat acute sciatic nerve injury model	PRP	Curcumin	Local PRP plus intraperitoneal curcumin as adjuvants to nerve repair	Repair only; repair + PRP; repair + curcumin

Abbreviations: PRF, platelet-rich fibrin; PRP, platelet-rich plasma; FD-PRP, freeze-dried platelet-rich plasma; L-PRF, leukocyte platelet-rich fibrin; H-PRF, horizontal platelet-rich fibrin; hASCs, human adipose-derived stem cells; STZ, streptozotocin; ROS, reactive oxygen species; pH, potential hydrogen; CMCS, carboxymethyl chitosan; Odex, oxidized dextran; OPC, oligomeric procyanidins; NLCs, nanostructured lipid carriers.

**Table 3:** Main findings of the included studies.

Study	Main Outcome(s)	Interpretation Within This Review
Zheng et al., 2019 [28]	FD-PRP provided sustained release of icariin and improved new bone formation, fibrocartilage regeneration, failure load, and stiffness	Strongest direct evidence that PRP served as a carrier for a natural compound
Zhao and Yuan, 2024 [29]	EOPM hydrogel showed antibacterial, antioxidant, injectable, and self-healing properties and accelerated infected diabetic wound repair	PRP was embedded within a natural-compound-enriched hydrogel system; this was a hybrid carrier design
Ghufran et al., 2020 [30]	Cur-hASCs + PRP improved wound closure, angiogenesis, fibroblast proliferation, and healing-marker expression	PRP acted more as a supportive regenerative matrix than a formal carrier
Murgia et al., 2020 [34]	Curcumin accumulation and metronidazole permeation through L-PRF and buccal tissue were demonstrated; nanocomposite was cytocompatible	PRF was part of a combined delivery platform rather than the sole carrier
Wafy et al., 2026 [32]	PRP + nano-propolis improved oxidative-stress modulation, collagen maturation, and tissue regeneration in canine wounds	Adjunctive synergistic regenerative use rather than direct carrier testing
Wang et al., 2025 [35]	TA-modified H-PRF had improved strength, reduced porosity, prolonged degradation, better bacterial exclusion, and good cytocompatibility	Natural compound modified PRF membrane performance and durability
Haghparast-Kenarsari et al., 2024 [38]	TA crosslinking reduced swelling and degradation and increased Young’s modulus without cytotoxicity	PRF scaffold properties were enhanced by a natural polyphenol crosslinker
He et al., 2024 [31]	COO@PRP hydrogel showed ROS/pH-responsive sustained GF release and faster wound closure with reduced inflammation and increased VEGF/angiogenesis	PRP was delivered within a natural polyphenol-containing smart hydrogel
Bora et al., 2026 [36]	PRF + propolis improved recession depth reduction and keratinized tissue gain versus PRF alone	Early clinical evidence for propolis as a PRF adjunct in periodontal plastic surgery
Balani et al., 2024 [37]	PRF + MO and PRF + SIM both reduced crestal bone loss versus PRF alone; PRF + MO was comparable to PRF + SIM	Clinical evidence suggested possible benefit of Moringa oleifera as a PRF adjunct; however, the botanical intervention was insufficiently characterized, as the extract type, plant part, preparation method, and mode of incorporation into PRF were not reported.
Zavala et al., 2023 [33]	PRP + curcumin produced the best functional, electrophysiological, and histologic nerve-regeneration outcomes	Supportive evidence for combination therapy; not a formal PRP carrier study

Abbreviations: COO@PRP, CMCS/Odex/OPC/PRP hydrogel; Cur-hASCs, curcumin-preconditioned human adipose-derived stem cells; EOPM, [add full name if known]; FD-PRP, freeze-dried platelet-rich plasma; GF, growth factor; H-PRF, horizontal platelet-rich fibrin; L-PRF, leukocyte platelet-rich fibrin; MO, *Moringa oleifera*; pH, potential hydrogen; PRF, platelet-rich fibrin; PRP, platelet-rich plasma; ROS, reactive oxygen species; SIM, simvastatin; TA, tannic acid; VEGF, vascular endothelial growth factor.

**Table 4:** Risk of bias assessment of included studies.

Study	Design	Tool Applied	Overall Judgment	Rationale
Bora et al., 2026 [36]	Randomized clinical trial	RoB 2	Some concerns	The study reported random allocation with Random Allocation Software, allocation concealment with SNOSE, blinding of participants and outcome assessors, sample-size calculation, and complete 3-month follow-up with no losses. However, the methods section contained internal inconsistencies regarding group labeling and the timing/assignment of propolis irrigation, which introduced some concern regarding deviations from intended interventions and reporting clarity.
Balani et al., 2024 [37]	Non-randomized comparative human study	ROBINS-I	Serious risk of bias	Randomization, allocation concealment, and blinding were not reported. Group assignment appeared non-random and partly side-based, as the report stated that right-sided implants were assigned to the PRF group and left-sided implants to the SIM/PRF group, while the handling of the Moringa group was not clearly explained. These features created substantial risk of confounding, selection bias, and classification bias. In addition, the natural intervention was insufficiently described, because the type of Moringa oleifera extract, plant part, preparation method, and incorporation into PRF were not reported.
Zheng et al., 2019 [28]	Animal study (rabbit)	SYRCLE	Some concerns	Rabbits were randomly allocated into three groups, specimen numbers were determined by power analysis, specimens for imaging/histology were randomly selected, and semiquantitative histologic assessment was performed by two independent investigators. Nevertheless, allocation concealment, random housing, and blinding of surgeons/caregivers were not explicitly described.
Zavala et al., 2023 [33]	Animal study (rat)	SYRCLE	Some concerns	The rats were randomly allocated into four treatment groups and all animals completed the 12-week phase. Outcome collection was structured and included functional, electrophysiological, and histologic endpoints. However, no explicit blinding of outcome assessors or concealment procedures was reported in the retrieved methods text.
Ghufran et al., 2020 [30]	Animal study (rat) with in vitro component	SYRCLE for in vivo part	High risk	The in vivo study stated that diabetic rats were divided into four groups, but no randomization method, allocation concealment, or blinding of outcome assessment was reported. The in vivo sample was small, and the report provided limited detail on how histologic scoring was performed or whether assessors were masked.
He et al., 2024 [31]	Animal study (mouse) with in vitro component	SYRCLE for in vivo part	Some concerns	Mice were randomly divided into three groups, and outcome assessment included wound imaging, histology, and immunofluorescence. However, the study did not report blinding, concealment, or housing/allocation safeguards, and the reported wound-healing sample size was small.
Zhao and Yuan, 2024 [29]	Animal study (mouse) with in vitro component	SYRCLE for in vivo part	Some concerns	Diabetic infected mice were randomly divided into groups with n = 6, and wound area was measured digitally with Fiji. Histology and immunofluorescence were also performed. Still, no explicit blinding of treatment administration or outcome assessment was reported, and concealment procedures were not described.
Wafy et al., 2026 [32]	Animal study (dog)	SYRCLE	High risk	Wounds were randomly allocated within the same six dogs, but blinding was not reported and the design was vulnerable to unit-of-analysis problems because multiple wounds from the same animal were analyzed as treatment units. The authors themselves acknowledged that minor systemic influences across wounds could not be completely excluded. These features increased the risk of performance bias and pseudoreplication.
Murgia et al., 2020 [34]	In vitro/ex vivo formulation study	Not formally scored	Narrative appraisal only	This was a bench-scale nanocomposite and permeation study, not an interventional animal or clinical trial. Triplicate experiments and cytocompatibility testing were reported, but no formal randomization/blinding framework was applicable. The main limitations were exploratory design and limited external validity.
Haghparast-Kenarsari et al., 2024 [38]	In vitro scaffold study	Not formally scored	Narrative appraisal only	This was an in vitro scaffold optimization study using different tannic acid concentrations with physical testing, cytotoxicity, DAPI, and antibacterial assays. A formal RoB tool was not applicable, but the study remained vulnerable to selective outcome reporting and limited translational generalizability.
Wang et al., 2025 [35]	Ex vivo donor-derived membrane study	Not formally scored	Narrative appraisal only	This was a donor-derived H-PRF membrane study in which same-donor comparisons and triplicate experiments were performed. Although bench methods were clearly described, formal randomization and assessor blinding were not reported, and the study remained a preclinical material investigation with limited external validity.

Abbreviations: DAPI, 4′,6-diamidino-2-phenylindole; H-PRF, horizontal platelet-rich fibrin; PRF, platelet-rich fibrin; PRP, platelet-rich plasma; RoB, risk of bias; RoB 2, revised Cochrane risk-of-bias tool for randomized trials; ROBINS-I, Risk Of Bias In Non-randomized Studies of Interventions; SIM, simvastatin; SNOSE, sequentially numbered, opaque, sealed envelopes; SYRCLE, Systematic Review Centre for Laboratory Animal Experimentation risk-of-bias tool.

## Data Availability

No new data were created or analyzed in this study. Data sharing is not applicable to this article.

## Supplementary Materials

The following supporting information can be downloaded at: https://www.mdpi.com/article/10.3390/ma19142970/s1.

## Abbreviations

The following abbreviations are used in this manuscript:

A-PRF	advanced platelet-rich fibrin
AL-PRF	antibiotic-loaded platelet-rich fibrin
AMC	amoxicillin/clavulanic acid
APC	autologous platelet concentrate
AUC	area under the curve
c-PRF	concentrated platelet-rich fibrin
CGF	concentrated growth factor
CMAP	compound muscle action potential
CMCS	carboxymethyl chitosan
COO@PRP	CMCS/Odex/OPC/PRP hydrogel
EOPM	metal-–phenol nanoparticle-containing PRP hydrogel formulation
FD-PRP	freeze-dried platelet-rich plasma
FGF	fibroblast growth factor
GF	growth factor
H-PRF	horizontal platelet-rich fibrin
hASCs	human adipose-derived stem cells
HGF-1	human gingival fibroblast cell line
i-PRF	injectable platelet-rich fibrin
L-PRF	leukocyte platelet-rich fibrin
LPCGF	liquid-phase concentrated growth factor
MO	Moringa oleifera
NLCs	nanostructured lipid carriers
Odex	oxidized dextran
OPC	oligomeric procyanidins
PDGF	platelet-derived growth factor
PICOS	Population, Intervention, Comparator, Outcomes, Study design
PRF	platelet-rich fibrin
PRP	platelet-rich plasma
RD	recession depth
ROBINS-I	Risk Of Bias In Non-randomized Studies of Interventions
RoB 2	revised Cochrane risk-of-bias tool for randomized trials
ROS	reactive oxygen species
SFI	sciatic functional index
SIM	simvastatin
STZ	streptozotocin
SYRCLE	Systematic Review Centre for Laboratory Animal Experimentation
TA	tannic acid
TGF-β1	transforming growth factor beta 1
VEGF	vascular endothelial growth factor
WKT	width of keratinized tissue
