# Supplementary material for: Use of Platelet-Rich Fibrin and Platelet-Rich Plasma as Delivery Systems for Natural Compounds: A Systematic Review
Source: Materials (Basel). 2026 Jul 10;19(14):2970. doi: 10.3390/ma19142970 (PMC13412838; doi:10.3390/ma19142970)
Supplement: Supplementary file 1 [file materials-19-02970-s001.zip › materials-4371374-supplementary.pdf]

PRISMA 2020 Checklist

Revised location/comment fields after editor comments

Section and Topic	Item #	Checklist item	Location where item is reported / revised comment
**TITLE**			
Title	1	Identify the report as a systematic review.	Title page: title identifies the report as a systematic review.
**ABSTRACT**			
Abstract	2	See the PRISMA 2020 for Abstracts checklist.	Abstract: structured Background/Objectives, Methods, Results and Conclusions. The abstract should keep the conclusion cautious, explicitly stating that evidence is heterogeneous, largely preclinical and not meta-analyzed.
**INTRODUCTION**			
Rationale	3	Describe the rationale for the review in the context of existing knowledge.	Section 1 Introduction: rationale for natural compounds, PRP/PRF and delivery-system need.
Objectives	4	Provide an explicit statement of the objective(s) or question(s) the review addresses.	End of Section 1 Introduction; Section 2.2 Review question.
**METHODS**			
Eligibility criteria	5	Specify the inclusion and exclusion criteria for the review and how studies were grouped for the syntheses.	Sections 2.3 PICOS framework, 2.4 Eligibility criteria and 2.9 Data synthesis.
Information sources	6	Specify all databases, registers, websites, organisations, reference lists and other sources searched or consulted to identify studies. Specify the date when each source was last searched or consulted.	Section 2.5 Information sources and search strategy; Table 1.
Search strategy	7	Present the full search strategies for all databases, registers and websites, including any filters and limits used.	Section 2.5 Information sources and search strategy; Table 1 gives full PubMed, Scopus and Embase searches.
Selection process	8	Specify the methods used to decide whether a study met the inclusion criteria of the review, including how many reviewers screened each record and each report retrieved, whether they worked independently, and if applicable, details of automation tools used in the process.	Section 2.6 Study selection.
Data collection process	9	Specify the methods used to collect data from reports, including how many reviewers collected data from each report, whether they worked independently, any processes for obtaining or confirming data from study investigators, and if applicable, details of automation tools used in the process.	Section 2.7 Data extraction.
Data items	10a	List and define all outcomes for which data were sought. Specify whether all results that were compatible with each outcome domain in each study were sought (e.g. for all measures, time points, analyses), and if not, the methods used to decide which results to collect.	Section 2.3 Outcomes; Section 2.7 Data extraction.
	10b	List and define all other variables for which data were sought (e.g. participant and intervention characteristics, funding sources). Describe any assumptions made about any missing or unclear information.	Section 2.7 Data extraction; unclear/missing intervention details noted in Sections 3.5 and 4.3.
Study risk of bias assessment	11	Specify the methods used to assess risk of bias in the included studies, including details of the tool(s) used, how many reviewers assessed each study and whether they worked independently, and if applicable, details of automation tools used in the process.	Section 2.8 Risk of bias assessment; Section 3.7 and Table 4. Clarifies that judgments were based on design/reporting domains, not on statistical significance or direction of treatment effects.
Effect measures	12	Specify for each outcome the effect measure(s) (e.g. risk ratio, mean difference) used in the synthesis or presentation of results.	No pooled effect measures were prespecified or calculated because the evidence was too heterogeneous for meta-analysis. Individual study outcomes were summarized descriptively/narratively in Sections 3.3-3.6 and Table 3; no summary effect estimates or confidence intervals should be inferred from the review.
Synthesis methods	13a	Describe the processes used to decide which studies were eligible for each synthesis (e.g. tabulating the study intervention characteristics and comparing against the planned groups for each synthesis (item #5)).	Sections 2.3, 2.4 and 2.9; Section 3.3 Synthesis of the included evidence. Studies were grouped by platelet concentrate, natural compound, carrier/scaffold role, application model and study design.
	13b	Describe any methods required to prepare the data for presentation or synthesis, such as handling of missing summary statistics, or data conversions.	No meta-analytic data preparation, imputation of missing summary statistics or numerical data conversion was performed. Data were extracted and tabulated descriptively. This should remain explicitly stated because the available studies did not provide comparable outcome metrics suitable for pooling.
	13c	Describe any methods used to tabulate or visually display results of individual studies and syntheses.	Sections 2.9 and 3; Figure 1; Tables 2-4. Table 3 summarizes individual study findings and interpretation within the narrative synthesis; Table 4 summarizes risk of bias/methodological appraisal.
	13d	Describe any methods used to synthesize results and provide a rationale for the choice(s). If meta-analysis was performed, describe the model(s), method(s) to identify the presence and extent of statistical heterogeneity, and software package(s) used.	Section 2.9 Data synthesis: narrative synthesis only. Rationale: marked heterogeneity in study design, experimental model, target tissue, platelet-concentrate preparation, natural compound/formulation, comparator groups, outcomes and follow-up. No meta-analysis model, statistical heterogeneity measure (I2/tau2/Q) or meta-analysis software was used.

Section and Topic	Item #	Checklist item	Location where item is reported / revised comment
	13e	Describe any methods used to explore possible causes of heterogeneity among study results (e.g. subgroup analysis, meta-regression).	No formal subgroup analysis or meta-regression was conducted because no meta-analysis was feasible and the included evidence base was small and heterogeneous. Heterogeneity was explored narratively by study category, platelet concentrate type, natural compound, mode of incorporation/delivery, application model and study design in Sections 2.9 and 3.2-3.6, with limitations discussed in Section 4.3.
	13f	Describe any sensitivity analyses conducted to assess robustness of the synthesized results.	No formal sensitivity analysis was conducted. Robustness of the narrative synthesis was not statistically tested; therefore, conclusions are framed as preliminary and hypothesis-generating. This limitation should be stated explicitly in Section 4.3 and reflected in the Abstract/Conclusions.
Reporting bias assessment	14	Describe any methods used to assess risk of bias due to missing results in a synthesis (arising from reporting biases).	No formal reporting-bias or small-study-effects assessment was conducted (e.g. funnel plot or regression test) because no pooled synthesis was performed and the studies were few, heterogeneous and mostly preclinical. This methodological limitation should be explicitly acknowledged in Section 4.3.
Certainty assessment	15	Describe any methods used to assess certainty (or confidence) in the body of evidence for an outcome.	No formal outcome-level certainty framework (e.g. GRADE) was applied. Certainty/confidence is discussed narratively only, based on predominance of preclinical/bench studies, small samples, heterogeneity and risk-of-bias concerns. Add or retain a clear statement in Methods/Discussion that no formal GRADE rating was performed and that overall certainty is low/very low.
**RESULTS**			
Study selection	16a	Describe the results of the search and selection process, from the number of records identified in the search to the number of studies included in the review, ideally using a flow diagram.	Section 3.1 Study selection; Figure 1 PRISMA 2020 flow diagram.
	16b	Cite studies that might appear to meet the inclusion criteria, but which were excluded, and explain why they were excluded.	Partially reported in Figure 1 with exclusion categories; individual excluded studies were not cited. If requested by the journal, add a short excluded-studies table or supplementary list with reasons.
Study characteristics	17	Cite each included study and present its characteristics.	Section 3.2 Study characteristics; Table 2.
Risk of bias in studies	18	Present assessments of risk of bias for each included study.	Section 3.7 Risk of bias assessment; Table 4.
Results of individual studies	19	For all outcomes, present, for each study: (a) summary statistics for each group (where appropriate) and (b) an effect estimate and its precision (e.g. confidence/credible interval), ideally using structured tables or plots.	Sections 3.4-3.6 and Table 3 present individual study findings descriptively. Complete structured effect estimates and precision measures are not available/reported consistently across the included studies; this absence should be treated as a limitation and not replaced by pooled estimates.
Results of syntheses	20a	For each synthesis, briefly summarise the characteristics and risk of bias among contributing studies.	Sections 3.3-3.7; Tables 3-4.
	20b	Present results of all statistical syntheses conducted. If meta-analysis was done, present for each the summary estimate and its precision (e.g. confidence/credible interval) and measures of statistical heterogeneity. If comparing groups, describe the direction of the effect.	No statistical synthesis or meta-analysis was conducted. Direction of findings is described qualitatively in Sections 3.3-3.6 and Table 3. No pooled summary estimates, confidence intervals or statistical heterogeneity measures are available.
	20c	Present results of all investigations of possible causes of heterogeneity among study results.	No formal heterogeneity investigation was conducted. Heterogeneity is described narratively by study model, formulation, comparator, outcome and application area in Sections 3.2-3.6 and Section 4.3.
	20d	Present results of all sensitivity analyses conducted to assess the robustness of the synthesized results.	No sensitivity analyses were conducted; robustness of the synthesis was not statistically evaluated. Interpret conclusions cautiously as narrative and preliminary.
Reporting biases	21	Present assessments of risk of bias due to missing results (arising from reporting biases) for each synthesis assessed.	No formal assessment of reporting bias due to missing results was presented. This should remain transparently stated because the absence of such assessment limits objectivity of the narrative conclusions.
Certainty of evidence	22	Present assessments of certainty (or confidence) in the body of evidence for each outcome assessed.	Partially addressed narratively in Sections 4.1 and 4.3. No formal outcome-level certainty assessment is presented. If a formal GRADE table is not added, state clearly that certainty was not rated and that confidence in conclusions is limited/low because of heterogeneity, small evidence base, preclinical dominance and risk-of-bias concerns.
**DISCUSSION**			
Discussion	23a	Provide a general interpretation of the results in the context of other evidence.	Sections 4.1-4.2 and 4.4.
	23b	Discuss any limitations of the evidence included in the review.	Section 4.3 Limitations. Strengthen the wording to explicitly connect limitations to credibility: heterogeneous preclinical evidence, small clinical evidence base, moderate-to-high risk of bias and absence of formal certainty rating limit the objectivity and clinical strength of conclusions.
	23c	Discuss any limitations of the review processes used.	Section 4.3 Limitations. Strengthen by explicitly listing: no meta-analysis, no statistical heterogeneity testing, no sensitivity analysis, no reporting-bias assessment, no formal GRADE/outcome-level certainty assessment, and potential subjectivity inherent to narrative synthesis.
	23d	Discuss implications of the results for practice, policy, and future research.	Sections 4.5 Implications for practice and policy and 4.6 Implications for future research. Keep recommendations framed as investigational/research-oriented rather than practice-changing.
**OTHER INFORMATION**			

Section and Topic	Item #	Checklist item	Location where item is reported / revised comment
Registration and protocol	24a	Provide registration information for the review, including register name and registration number, or state that the review was not registered.	Section 2 Materials and Methods: systematic review registration.
	24b	Indicate where the review protocol can be accessed, or state that a protocol was not prepared.	Section 2 Materials and Methods: protocol/registration statement.
	24c	Describe and explain any amendments to information provided at registration or in the protocol.	Section 2 Materials and Methods: registration/protocol amendments statement.
Support	25	Describe sources of financial or non-financial support for the review, and the role of the funders or sponsors in the review.	Back matter: Funding statement reports no external funding. Add explicit statement that there was no funder/sponsor role, if not already present.
Competing interests	26	Declare any competing interests of review authors.	Back matter: Conflicts of Interest statement.
Availability of data, code and other materials	27	Report which of the following are publicly available and where they can be found: template data collection forms; data extracted from included studies; data used for all analyses; analytic code; any other materials used in the review.	Add a Data Availability statement. Suggested wording: "All data extracted and synthesized in this review are included in the article tables. No analytic code was generated because no statistical or meta-analytic synthesis was performed."

Source: PRISMA 2020 checklist. This version keeps the checklist transparent where analyses were not conducted.
